# High Breakdown Strength and Energy Storage Density in Aligned SrTiO_3_@SiO_2_ Core–Shell Platelets Incorporated Polymer Composites

**DOI:** 10.3390/membranes11100756

**Published:** 2021-09-30

**Authors:** Jie Chen, Xiaoyong Zhang, Xiao Yang, Chuanyang Li, Yifei Wang, Weixing Chen

**Affiliations:** 1 Shaanxi Key Laboratory of Optoelectronic Functional Materials and Devices, School of Materials Science and Chemical Engineering, Xi’an Technological University, Xi’an 710032, China; chenjie@xatu.edu.cn (J.C.); zhangxiaoyong371@163.com (X.Z.); chenwx@xatu.edu.cn (W.C.); 2 School of Electrical and Electronic Engineering, North China Electric Power University, Beijing 102206, China; yangxiao@ncepu.edu.cn; 3 Electrical Insulation Research Center, Institute of Materials Science, University of Connecticut, Storrs, CT 06269, USA; chuanyang.li@uconn.edu

**Keywords:** SrTiO_3_ platelets, composites, electric displacement, breakdown strength, electrical energy storage

## Abstract

Dielectric nanocomposites with high energy storage density (*U_e_*) have a strong attraction to high-pulse film energy-storage capacitors. Nevertheless, low breakdown strengths (*E_b_*) and electric displacement difference (*D_max_-D_rem_*) values of nanocomposites with incorporating the randomly distributed high dielectric constant additions, give rise to low *U_e_*, thereby hindering the development of energy-storage capacitors. In this study, we report on newly designed SrTiO_3_@SiO_2_ platelets/PVDF textured composites with excellent capacitive energy storage performance. SrTiO_3_@SiO_2_ platelets are well oriented in the PVDF when perpendicular to the electric field with the assistance of shear force in the flow drawing process to establish microscopic barriers in an inorganic–polymer composite that is able to substantially improve the *E_b_* of composites and enhance the *U_e_* accordingly. Finite element simulation demonstrates that the introduction of the highly insulating SiO_2_ coating onto the SrTiO_3_ platelets effectively alleviates the interface dielectric mismatch between filler and PVDF matrix, resulting in a reduction in the interface electric field distortion. The obtained composite film with optimized paraelectric SrTiO_3_@SiO_2_ platelets (1 vol%) exhibited a maximum *D_max_-D_rem_* value of 9.14 μC cm^−2^ and a maximum *U_e_* value of 14.4 J cm^−3^ at enhanced *E_b_* of 402 MV m^−1^, which are significantly superior to neat PVDF and existing dielectric nanocomposites.

## 1. Introduction

Polymer-based electrostatic capacitors have been widely utilized in electrical pulsed systems due to their high power density (MW), fast discharge time (μs), and long life-span [1]. The development of electrostatic capacitors is associated with the capacitive capabilities of dielectrics [2]. According to the energy storage density (*U_e_*) calculation formula (*U_e_ = 0.5ε_0_ε_r_E_b_*^2^, in the case of linear dielectrics), high *U_e_* could be realized by improving breakdown strength (*E_b_*) and enhancing the relative dielectric constant (*ε_r_*) simultaneously [3,4]. For instance, even at an ultra-high *E_b_* of 640 MV m^−1^, the *U_e_* (4–5 J cm^−3^) of existing commercial biaxially oriented polypropylene (BOPP) film is distinctly lower than currently supercapacitors (20–29 J cm^−3^) and batteries (200–2500 J cm^−3^), which is attributed to its low intrinsic *ε_r_* value of ~2.2 at 1 kHz, demonstrating a nonpolar characteristic [5,6,7]. 

Recently, the construction of nanocomposite films combined the respective merits of fillers with high-*ε_r_* and polymers with high-*E_b_* [8,9,10]. As a series of polar polymers, poly(vinylidene fluoride) (PVDF) and its copolymers (e.g. P(VDF-HFP)), can offer a relatively higher *ε_r_* (10–20 at 1 kHz) and a high *E_b_* (300–400 MV m^−1^), which could increase the *U_e_* (~10 J cm^−3^) in comparison with non-polar polymers (e.g. BOPP) [11]. Unfortunately, the obtained *ε_r_* value of most nanocomposites is increased at a cost of the serious reduction in *E_b_* by forming continuously conductive paths and depressing *U_e_* [12]. In addition, the additive concentrations of high-*ε_r_* inorganic nanofillers (i.e., >50 vol%) are inevitably introduced, thus causing limited *U_e_*, poor polymer matrix flexibility and high energy loss. This issue can be further addressed by designing filler–polymer interface structures and the effect of filler morphologies. Distinct from the 0D nanoparticles and 1D nanowires/nanofibers/nanotubes, 2D nanosheets/nanoclays with higher lateral size are more successful at achieving notably improved *E_b_* through constructing efficient conduction barriers [13,14]. Moreover, the lower additive concentrations of 2D nanosheets/nanoclays are added to achieve an enhanced *E_b_* of nanocomposites without the cost of *ε_r_* in comparison with the 0D nanoparticles and 1D nanowires/nanofibers/nanotubes. Meanwhile, the results of phase-field simulations confirm that dispersed parallel nanosheets in the polymer matrix is the most effective method to reduce the inhomogeneity of local electric field distribution [15,16]. Nevertheless, the most widely utilized 2D fillers, hexagonal boron nitride nanosheets (BNNS) (*ε_r_* = 3–5) and γ-Al_2_O_3_ (*ε_r_* = 9–10), have intrinsic low *ε_r_* and give rise to the limited *ε_r_* of nanocomposites [17,18]. Moreover, small-size nanosheets cannot be arranged in order along the vertical electric field direction by the flow extension method, which limits the further improvement of the *E_b_* and *U_e_* of the composites.

Herein, we propose a feasible strategy to construct a ferroelectric polymer-based composite incorporating a SrTiO_3_ paraelectric filler with a large size (diameter~1–5 μm). Unlike the high-*ε_r_* ferroelectric dielectrics such as BaTiO_3_ (*ε_r_* = 1000) ceramic whose *E_b_* decreases dramatically, the presence of moderate-*ε_r_* strontium titanate SrTiO_3_ (*ε_r_*~300 at 1 kHz) and low-*ε_r_* silicon oxide SiO_2_ (*ε_r_*~3.9 at 1 kHz) is beneficial to mitigate the dielectric constant gradient between the filler and the PVDF (*ε_r_*~10 at 1 kHz) matrix, thereby increasing *E_b_* due to the inhibition of interface field distortion [19,20]. It is more encouraging that well-oriented ST@SiO_2_ was achieved along a direction perpendicular to the electric field in the polymer matrix, which can also substantially improve the *E_b_* and *U_e_*. As a result, concurrently enhanced capacitive performance is endowed in composite films containing optimized ST@SiO_2_ platelets. The composite film incorporated with 1 vol% ST@SiO_2_ platelets delivers a high *D_max_-D_rem_* of 9.14 μC cm^−2^ at a high *E_b_* of 402 MV m^−1^, thereby achieving a maximum *U_e_* of 14.4 J cm^−3^, which is ≈115% greater than that (6.7 J cm^−3^) of PVDF at *E_b_* of 330 MV m^−1^ and represents the highest value ever reported for dielectric nanocomposites at respective breakdown strengths. This work provides a pathway to effectively enhance the energy storage capability of polymer composites by incorporating core–shell microscale 2D fillers. 

Herein, we propose a feasible strategy to construct a ferroelectric polymer-based composite incorporating a SrTiO_3_ paraelectric filler with a large size (diameter~1–5 μm). Unlike the high-*ε_r_* ferroelectric dielectrics such as BaTiO_3_ (*ε_r_* = 1000) ceramic whose *E_b_* decreases dramatically, the presence of moderate-*ε_r_* strontium titanate SrTiO_3_ (*ε_r_*~300 at 1 kHz) and low-*ε_r_* silicon oxide SiO_2_ (*ε_r_*~3.9 at 1 kHz) is beneficial to mitigate the dielectric constant gradient between the filler and the PVDF (*ε_r_*~10 at 1 kHz) matrix, thereby increasing *E_b_* due to the inhibition of interface field distortion [19,20]. It is more encouraging that well-oriented ST@SiO_2_ was achieved along a direction perpendicular to the electric field in the polymer matrix, which can also substantially improve the *E_b_* and *U_e_*. As a result, concurrently enhanced capacitive performance is endowed in composite films containing optimized ST@SiO_2_ platelets. The composite film incorporated with 1 vol% ST@SiO_2_ platelets delivers a high *D_max_-D_rem_* of 9.14 μC cm^−2^ at a high *E_b_* of 402 MV m^−1^, thereby achieving a maximum *U_e_* of 14.4 J cm^−3^, which is ≈115% greater than that (6.7 J cm^−3^) of PVDF at *E_b_* of 330 MV m^−1^ and represents the highest value ever reported for dielectric nanocomposites at respective breakdown strengths. This work provides a pathway to effectively enhance the energy storage capability of polymer composites by incorporating core–shell microscale 2D fillers. 

## 2. Materials and Methods

### 2.1. Synthesis of SrTiO_3_@SiO_2_ Platelets

SrTiO_3_ (ST) platelets with a large average size were synthesized using a previously reported two-step molten salt method [21]. The pre-prepared Bi_4_Ti_3_O_12_ precursor and SrCO_3_ powders were weighed in 1:10 molar ratios and stirred ultrasonically for 4 h to promote the diffusion of Sr^2+^ ions into Bi_4_Ti_3_O_12_ cells. The above mixture and NaCl was weighted in a 1:1 mass ratio and stirred for 4 h by sonication. The mixture was heated at 1000 °C/ 2h to obtain SrTiO_3_ plates. The reaction mechanism of preparing the SrTiO_3_ plates was as follows:

(1) $${\;\mathrm{Bi}}_{4}{\mathrm{Ti}}_{3}O_{12}+{\mathrm{SrCO}}_{3}\to {\mathrm{SrTiO}}_{3}+{\mathrm{Bi}}_{2}O_{3}+{\mathrm{CO}}_{2}$$

The synthesized SrTiO_3_ plates were washed repeatedly with dilute hydrochloric acid and deionized water for 2 h. In order to confirm the absence of Cl^−^, AgNO_3_ solution was used during the washing procedure. The ST platelets were coated with a SiO_2_ layer as a result of the hydrolysis reaction of the tetraethoxysilane (TEOS, 28%). First, a certain number of ST platelets and PVP powders were dispersed in 200 ml ethyl alcohol and stirred for 60 min, then homogeneous mix dispersion was obtained by ultrasonic dispersion. Second, TEOS (28%) was added to ethyl alcohol and stirred for 60 min (designated A). Meanwhile, the deionized water was introduced into ethyl alcohol, and ammonia water was added to the above solution to regulate the pH (designated B). Next, the A solution was added to the B solution by inches and stirred for 1 h (designated C). Subsequently, the C solution was added slowly, drop by drop, into the ST suspension solution. Finally, the SrTiO_3_@SiO_2_ platelets were obtained by centrifugation washed six times with deionized water, and then calcinated at 800 °C for 2 h.

### 2.2. Fabrication of the Composites

**Figure 1:**
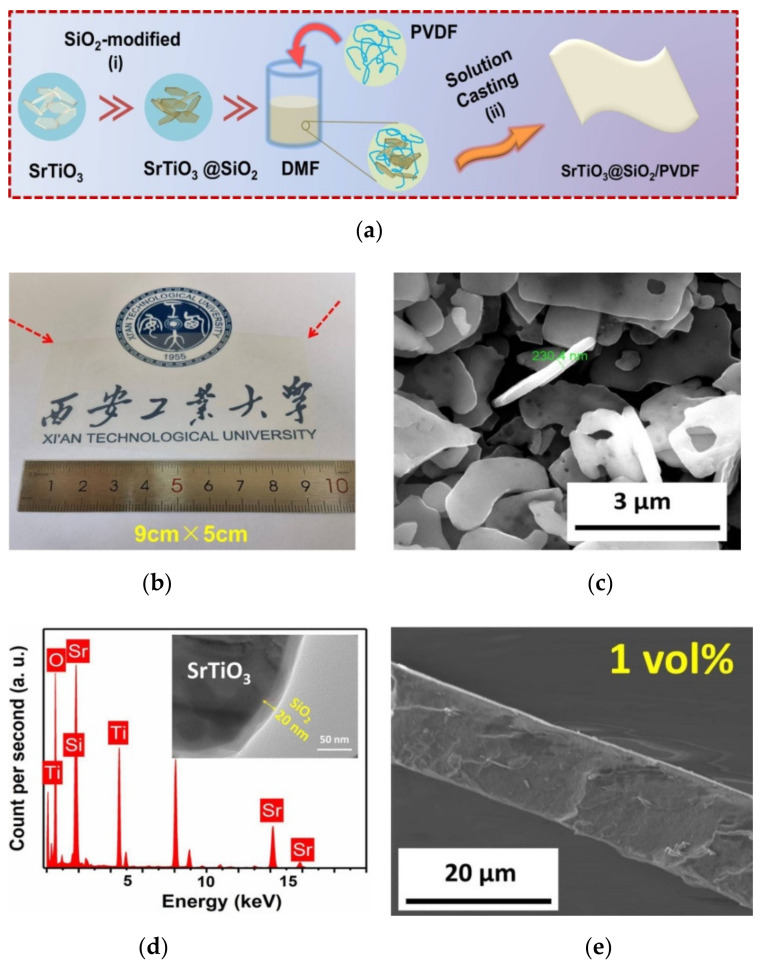
(**a**) Schematic illustration of the synthesis of plate-like SrTiO_3_@SiO_2_ powders and the manufacturing process of composites by solution casting process. (**b**) Sample optical image of a composite with a 1 vol% SrTiO_3_@SiO_2_ platelet. (**c**) SEM image of SrTiO_3_@SiO_2_ platelets. (**d**) EDS spectrum of SrTiO_3_@SiO_2_ platelets, inset shows TEM morphology of core–shell structure platelets. (**e**) Cross-section SEM images of composites with 1 vol% platelets.

The preparation procedure is shown in Figure 1a. The composites were fabricated using the solution casting method through tuning the ST@SiO_2_ platelet contents (1~4 vol%). PVDF was used as the polymer matrix of the composites. First, a certain number of ST@SiO_2_ platelets were dispersed in DMF (10 mL, Letai Co., China) with the help of ultrasonic dispersion for 15 min and continuously stirred for 24 h to form ST@SiO_2_/DMF suspension. Then, 1 g PVDF (Alfa Aesar) powders were proportionally dissolved in the suspension solution and stirred, uninterrupted, for 24 h to give ST@SiO_2_/PVDF suspension. Subsequently, the ST@SiO_2_/PVDF suspension was cast on the transparent glass substrate by a solution casting machine (MSK-AFA-L800, Shenyang Kejing Automation Equipment Co., LTD, Shenyang, China) at a casting rate of 15 mm/s and heated at 60 °C/ 0.5 h in a vacuum oven. Finally, in order to densify the composites, they were placed at 200 °C/5 min in a vacuum oven and then quickly quenched in deionized ice water. The composites were heated at 60 °C/24 h. 

### 2.3. Characterization

The microstructural morphology of the ST@SiO_2_ plates and composites was obtained by conducting scanning electron microscopy (SEM, Quanta FEG400, FEI, Ltd., Pittsburgh, PA, USA). The composites were fractured after being frozen in liquid nitrogen to observe their cross-section morphology. The crystallographic phase structure of the composites was created by X-ray diffraction (XRD; X’pert PRO, Panalytical, EA Almelo, Holland). Two sides of the composites with platinum electrodes (2 mm diameter, 100 nm thickness) were sputtered using the Hitachi Ion Sputter (MC1000, Hitachi High-Tech, Tokyo, Japan) for the dielectric and energy storage measurements. The frequency dependencies of dielectric performance were collected using an Agilent impedance analyzer (4294A, Keysight (Agilent) Technologies, Santa Clara, CA, USA) in a frequency range of 1 kHz to 20 MHz. The electrical displacement-field strength (*D-E*) loops and energy storage properties were collected employing a ferroelectric tester (RTI-Premier II, Radiant Technologies, Lewis Center, OH, USA) at 10 Hz. The DC electric resistivity and leakage current density were employed using *I-V* measurement (Poly*K* Technologies, Philipsburg, PA, USA). Finite element simulations and electric current interface in AC/DC models were used to obtain the distribution of the local electric field in the composite films. The electric distribution was calculated by the Poisson equation for electrostatic field. A positive DC voltage of 5 kV was applied on the top boundary, and the bottom boundary was grounded. Free triangle mesh was created in the model. In the simulation system, the electric conductivity of SrTiO_3_, SiO_2_ and PVDF was set as 10^−7^ S/m, 10^−14^ S/m, and 5×10^−10^ S/m, respectively.

## 3. Results and Discussion

### 3.1. Structural and Morphology Characterization 

The ST@SiO_2_ platelets/PVDF composites were produced by a well-prepared solution casting process, as presented in Figure 1a. The high-quality composites had a large film size of 45, 30 and 40 cm^2^, respectively, as seen in the optical photos of Figure 1b and Figure S1. Figure S2 presents the HRTEM pattern of the SrTiO_3_ platelets. It is clear that the lattice fringes were straight and parallel to each other, indicating that the as-prepared SrTiO_3_ platelets crystallized well. Figure 1c shows the SEM photo of ST@SiO_2_ platelets. It is obvious that the ST platelets had an average thickness of 200–400 nm and a diameter of 1–5 μm, respectively. As displayed in Figure 1c, the observable phase boundary between the SiO_2_ phase and the ST crystal phase was determined and the SiO_2_ layer had an average thickness of 20 nm. The presence of silicon from the energy-dispersive X-ray spectroscopy (EDS) spectrum of transmission electron microscopy (TEM) verified the successful coating of a SiO_2_ layer on the ST platelets, as observed in the inset of Figure 1c. Composite films had an average thickness of approximately 17–19 μm, as confirmed from the cross-section SEM photos (Figure 1d, Figure S3 and Figure S4). It is striking that the ST@SiO_2_ platelets were incorporated into the PVDF matrix due to the forceful combination between ST@SiO_2_ platelets and the PVDF matrix. In addition, most ST@SiO_2_ platelets were parallel to the composites during the casting process, which brought positive benefits to the dielectric property of the polymer matrix. 

The ST@SiO_2_ platelets/PVDF composites were produced by a well-prepared solution casting process, as presented in Figure 1a. The high-quality composites had a large film size of 45, 30 and 40 cm^2^, respectively, as seen in the optical photos of Figure 1b and Figure S1. Figure S2 presents the HRTEM pattern of the SrTiO_3_ platelets. It is clear that the lattice fringes were straight and parallel to each other, indicating that the as-prepared SrTiO_3_ platelets crystallized well. Figure 1c shows the SEM photo of ST@SiO_2_ platelets. It is obvious that the ST platelets had an average thickness of 200–400 nm and a diameter of 1–5 μm, respectively. As displayed in Figure 1c, the observable phase boundary between the SiO_2_ phase and the ST crystal phase was determined and the SiO_2_ layer had an average thickness of 20 nm. The presence of silicon from the energy-dispersive X-ray spectroscopy (EDS) spectrum of transmission electron microscopy (TEM) verified the successful coating of a SiO_2_ layer on the ST platelets, as observed in the inset of Figure 1c. Composite films had an average thickness of approximately 17–19 μm, as confirmed from the cross-section SEM photos (Figure 1d, Figure S3 and Figure S4). It is striking that the ST@SiO_2_ platelets were incorporated into the PVDF matrix due to the forceful combination between ST@SiO_2_ platelets and the PVDF matrix. In addition, most ST@SiO_2_ platelets were parallel to the composites during the casting process, which brought positive benefits to the dielectric property of the polymer matrix. 

**Figure 2:**
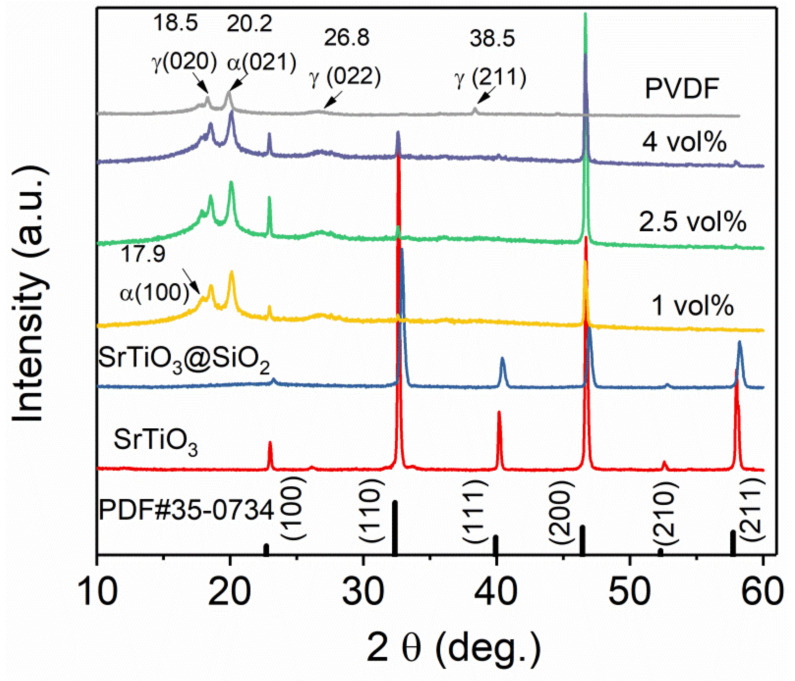
XRD patterns of SrTiO_3_, SrTiO_3_@SiO_2_, PVDF, and composites with SrTiO_3_@SiO_2_ platelets, respectively.

XRD patterns of ST, ST@SiO_2_ platelet, PVDF, and composites with SrTiO_3_@SiO_2_ platelets are presented in Figure 2. The perovskite structure was verified by the powder diffraction peaks of ST, ST@SiO_2_ platelets, and in line with the SrTiO_3_ standard card (PDF#35-0734). It can be seen that the XRD pattern of the SrTiO_3_@SiO_2_/PVDF composites contained the perovskite phase for SrTiO_3_@SiO_2_. Clearly, a preferred(200) direction orientation was obtained in all composites, indicating that most platelets were well orientated in the composites during the solution casting process, as verified by cross-section SEM photos. The composite films contained obvious non-polar α (100), α (021), γ (020), γ (022), and γ (211) phases of the PVDF matrix. The XRD spectra of all composites had a similar trend with that of the PVDF matrix, which proved that the phase transformation of the PVDF matrix could not be induced by the incorporation of a certain number of SrTiO_3_@SiO_2_ platelets. The non-polar γ phase was obtained during the quenching process, benefitting the energy storage abilities of composites. 

### 3.2. Weak-Field Dielectric Characteristics

**Figure 3:**
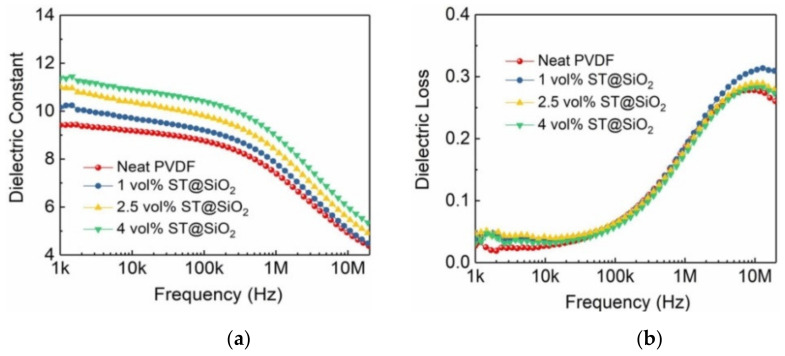
(**a**) Dielectric constant as a function of frequency, (**b**) dielectric loss as a function of frequency of PVDF and composites with SrTiO_3_@SiO_2_ platelets.

The dielectric properties as a function of frequency of pristine PVDF and composites with SrTiO_3_@SiO_2_ platelets are plotted in Figure 3. With increasing frequency, the dielectric constant of the composites was reduced. As expected, the incremental dielectric constant was obtained by increasing the SrTiO_3_@SiO_2_ loadings in composites. For instance, the dielectric constant increased from 9.41 for neat PVDF to 11.36 for the 4 vol% ST@ SiO_2_/PVDF composite (Figure 3a). As presented in Figure 3b, one peak was in the low-frequency (<1 kHz) range, representing α_a_ of the segmental motions in the PVDF amorphous phase [22]. The other peak was in the high-frequency (~10 MHz) range, expressing α_c_ relaxation of the PVDF crystalline phase [23]. Obviously, with an increased SrTiO_3_@SiO_2_ volume fraction, the low-frequency relaxation peak increased, whereas the high-frequency relaxation peak inversely decreased, which was closely associated with the more formed crystallization nuclei and incremental crystallinity of PVDF. Meanwhile, due to a relatively low dielectric constant (i.e., 3.9 at 1 kHz) of SiO_2_, the dielectric constant of the composite films with ST@SiO_2_ platelets was lower than that of the ST platelets counterparts. The superiority of the ST@SiO_2_ platelets over ST platelets was verified in respect to the repressive loss, as shown in Figure S5. For instance, dielectric loss decreased from 0.072 for the 4 vol% ST /PVDF composite to 0.035 for the 4 vol% ST@ SiO_2_/PVDF composite, as presented in Figure S6.

### 3.3. Weibull Breakdown Field Distribution

**Figure 4:**
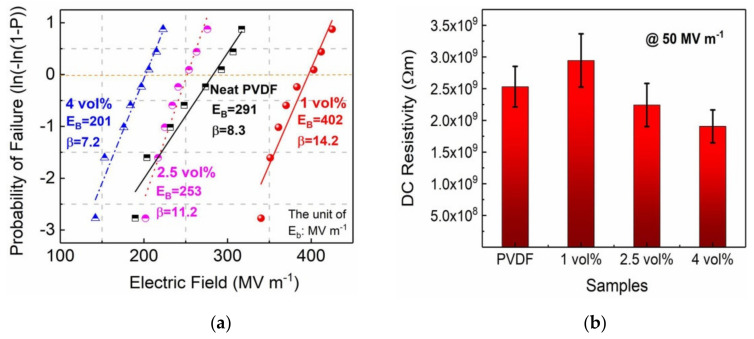
(**a**) Weibull breakdown field distribution, (**b**) DC electrical resistivity at 50 MV m^−1^ of PVDF and composites with SrTiO_3_@SiO_2_ platelets.

The Weibull distribution of PVDF and composites with SrTiO_3_@SiO_2_ platelets were analyzed by adopting the Weibull statistics [24]:

(2) $$P\;\left(E\right)=1-\exp\left[-{\left(\frac{E}{E_{b}}\right)}^{\beta }\right]$$

where *P(E)* is the probability of failure, *E* is the experimental electric breakdown strength, *E_b_* is the electric breakdown strength at *P(E)* of 63.2%, and *β* represents the reliability, as shown in Figure 4a. Notably, the 1 vol% ST@ SiO_2_/PVDF composite delivers a maximum *β* value of 14.2, indicating its high reliability. As plotted in Figure 4a, the 1 vol% ST@ SiO_2_/PVDF composite shows a larger *E_b_* value than that of PVDF, e.g., 402 MV m^−1^ of the 1 vol% film vs. 291 MV m^−1^ of the PVDF. Increasing the filler content to 4 vol% reduced *E_b_* to 201 MV m^−1^. Obviously, the *E_b_* is affected by optimized SrTiO_3_@SiO_2_ platelet contents, validated by DC electrical resistivity and *I-V* measurement, as presented in Figure 4b and Figure S7. The 1 vol% ST@ SiO_2_/PVDF composite has the highest DC electrical resistivity, e.g., 2.94 × 10^9^ Ω m of the 1 vol% film vs. 2.5 × 10^9^ Ω m of the PVDF film vs. 1.9 × 10^9^ Ω m of the 4 vol% film at 50 MV m^−1^. 

**Figure 5:**
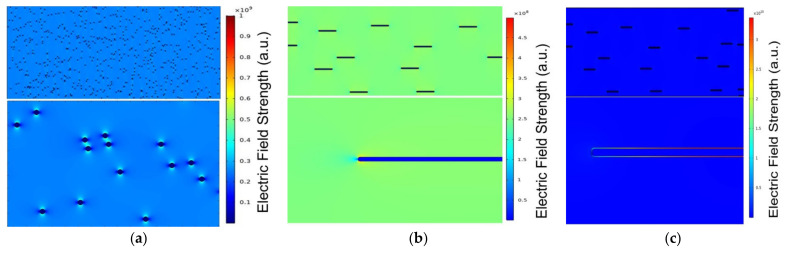
The distribution of the electric field simulated by finite element methods for the (**a**) SrTiO_3_ spheres /PVDF, (**b**) SrTiO_3_ platelets /PVDF, and (**c**) SrTiO_3_@SiO_2_ platelets /PVDF composites.

The electric field distribution in the SrTiO_3_ spheres /PVDF, SrTiO_3_ platelets /PVDF, and SrTiO_3_@SiO_2_ platelets /PVDF composites was analyzed by finite element simulation and presented in Figure 5 [25,26]. We selected the Y–Z section in a physical model, considering the orientation of platelets inside the PVDF matrix. The blue, green to red color scale bars represent the magnitude of electric field strength from low, medium to high electric field strength, respectively. A field strength of 200 MV m^−1^ was applied in the two-dimensional simulation system. It was clear that the electric field in the PVDF matrix was redistributed due to the dielectric constant mismatch between SrTiO_3_ and PVDF. For SrTiO_3_ spheres, the electric field was heavily distorted in the margin region along the electric field direction, caused by the accumulation of a great number of carriers (Figure 5a). When the spheres were replaced with platelets, the electric field distortion was alleviated, as shown in Figure 5b. In addition, with the introduction of the high-insulation SiO_2_ layer, the further suppressed local electric field of SrTiO_3_@SiO_2_ platelets was achieved (Figure 5c). The low-*ε_r_* silicon oxide SiO_2_ (*ε_r_*~3.9 at 1 kHz) was beneficial to mitigate the dielectric constant gradient between the filler and the PVDF (*ε_r_*~10 at 1 kHz) matrix, thereby increasing *E_b_* because of the inhibition of interface field distortion. The simulation results were in keeping with the experimental data, which theoretically revealed the immanent cause of the high *E_b_* of designed platelets core–shell structure.

### 3.4. Electrical Displacement

**Figure 6:**
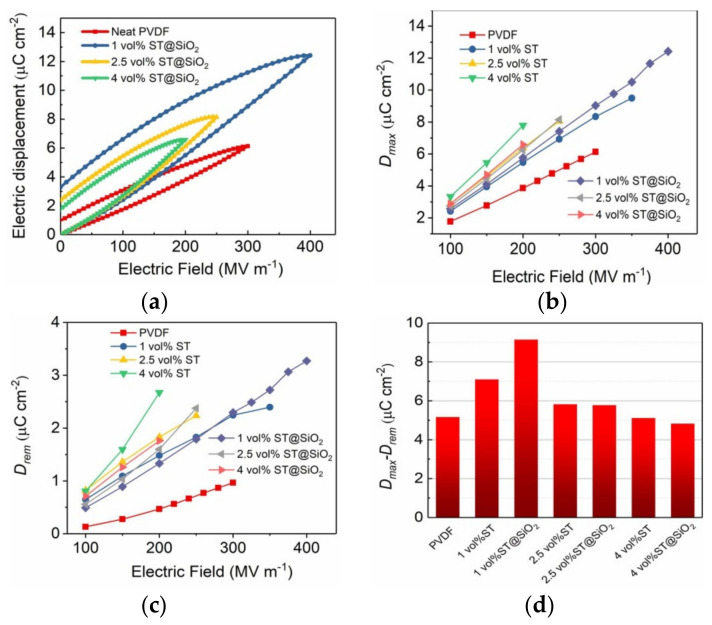
(**a**) Unipolar *D–E* loops at breakdown strengths. (**b**) Maximum displacement. (**c**) Remnant displacement and (**d**) electric displacement difference of PVDF and composites as a function of platelets content measured at varied electric fields.

*D–E* loops of PVDF and composites with SrTiO_3_@SiO_2_ platelets are characterized in Figure 6a and Figure S8. The *D_max_* and *D_rem_* are obtained from *D*–*E* loops, which are presented in Figure 6b,c. *D_max_* is the maximum electric displacement, *D_rem_* is the remnant electric displacement when removing the applied field. With the increase in the SrTiO_3_@SiO_2_ volume fraction, both *D_max_* and *D_rem_* increased, which is in line with the trend of a weak dielectric constant. On the basis of the calculation formula of energy storage density, substantially enhanced electric displacement difference (*D_max_-D_rem_*) values are thus indispensable to boost a high *U_e_* at high *E_b_*. Impressively, the highest *D_max_-D_rem_* value of 9.14 μC cm^−2^ was achieved in the 1 vol% SrTiO_3_@SiO_2_ /PVDF composite at an *E_b_* of 402 MV m^−1^, which was a ≈ 58% greater increment than 5.77 μC cm^−2^ of 1 vol% SrTiO_3_@SiO_2_ /PVDF composite at 250 MV m^−1^ and a ≈ 77% larger enhancement than 5.16 μC cm^−2^ of PVDF at 300 MV m^−1^, respectively, as revealed in Figure 6d. 

### 3.5. Capacitive Energy-Storage Capability 

**Figure 7:**
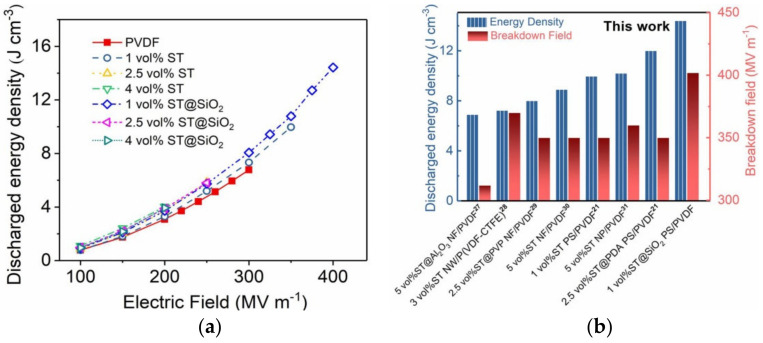
(**a**) Discharged energy density at varied electric fields of PVDF and composites as a function of platelets content measured at varied electric fields and (**b**) comparison of energy density and breakdown field between this contribution and previously reported references.

Based on the unipolar *D*–*E* loops of PVDF and composites as a function of platelets content measured at varied electric fields, the energy-storage performance (charged/discharged energy density, efficiency) is presented in Figure 7a, Figure S9, Figure S10, and Figure S11. Charged energy density (*U*) was obtained by calculating the integral area among the charged curve and the vertical axis; the *U_e_* was decided by the integration area among the discharged curve and the vertical axis. The increment in charged energy density was observed in Figure S10, in accordance with the enhancement of the *D_max_*. In comparison with the *U* of PVDF (9.8 J cm^−3^) at 300 MV m^−1^, the composite film (i.e*.,* 1 vol%) achieved a much higher *U* of 14.7 J cm^−3^. Intriguingly, the composite film with optimized SrTiO_3_@SiO_2_ platelets content (i.e*.,* 1 vol%) reached the maximum *U_e_* of 14.4 J cm^−3^, which was 115% greater than that (6.7 J cm^−3^) of pristine PVDF at *E_b_* of 330 MV m^−1^. The superior capacitive energy storage capabilities were ascribed to the highest *D_max_-D_rem_* value of 9.14 μC cm^−2^ at high *E_b_* of 402 MV m^−1^. 

The *U_e_* and *E_b_* of the ST@SiO_2_ platelets (ST@SiO_2_ PS)/PVDF with the available composites incorporating ST nanoparticles (NP), nanowires (NW), nanofibers (NF), platelets (ST PS), ST@Al_2_O_3_ nanofibers (ST@Al_2_O_3_ NF), ST@PVP nanofibers (ST@PVP NF), and ST@PDA platelets (ST@PDA PS) were comprehensively compared [21,27,28,29,30,31], as compared in Figure 7b. Evidently, the maximum *U_e_* of 14.4 J cm^−3^ accompanied by a high *E_b_* of 402 MV m^−1^ was obtained in the ST@SiO_2_ PS/PVDF composite film (1 vol%), outperforming the existing dielectric nanocomposites. For example, ST NP/PVDF, ST NW/P(VDF-CTFE), ST NF/PVDF, ST@Al_2_O_3_ NF/PVDF, and ST@PVP NF/PVDF possessed the *U_e_* of 10.2, 7.23, 8.9, 6.9 and 8 J cm^−3^ with breakdown strengths of 360, 370, 350, 312, 350 and 350 MV m^−1^, respectively. The superiority of ST@SiO_2_ PS/PVDF composite was also demonstrated in comparison to those of ST PS/PVDF (1 vol%) and ST@PDA PS/PVDF (1 vol%) composites that possessed the *U_e_* of 9.96 and 11.8 J cm^−3^ with respective breakdown strengths of 350 and 375 MV m^−1^, as listed in Table S1. These encouraging features exhibited that core–shell ST@SiO_2_ platelets/PVDF composites are highly efficient at achieving excellent energy storage performance. 

## 4. Conclusions

In this study, we presented newly designed ST@SiO_2_ platelets and a PVDF composite, which was obtained through the full-fledged solution-casting method. Paraelectric ST platelets were successfully synthesized using a molten salt process and coated by insulation SiO_2_. The outstanding combination of high dielectric constant, high electric displacement difference, breakdown strength, and greatly enhanced energy density of composites by incorporating ST@SiO_2_ platelet was attributed to the construction of the microscopic barrier layer and structural compatibility. According to the results of the distribution of the electric field simulated by finite element methods, ST@SiO_2_ platelets were the most effective filler in suppressing interface electric field distortion in comparison with untreated ST platelets and spheres, thereby greatly enhancing the breakdown strength and corresponding energy density. Ultimately, we achieved a maximal *D_max_-D_rem_* of 9.14 μC cm^−2^ and a *E_b_* of 402 MV m^−1^, resulting in a high *U_e_* of ≈14.4 J cm^−3^ in the 1 vol% ST@SiO_2_/PVDF composite, which was 115% greater than that (6.7 J cm^−3^) of PVDF at 330 MV m^−1^ and significantly superior to the *U_e_* at the respective *E_b_* of currently represented dielectric nanocomposites. This contribution verifies that a textured arrangement of optimized SrTiO_3_@SiO_2_ core–shell platelets greatly enhanced dielectric and energy storage abilities of polymer composites, which are attractive as candidates for commercial dielectric capacitors. Compared with benchmark BOPP, the discharged efficiency of SrTiO_3_@SiO_2_ platelets /PVDF textured composites with high energy density should be further improved to achieve a low energy loss, providing the possibility of implementing practical applications of textured composites.

## Data Availability

The data presented in this study are available on request from the corresponding author.

## Supplementary Materials

The following are available online at https://www.mdpi.com/article/10.3390/membranes11100756/s1, Figure S1: Cross-section SEM images of composites filled with 2.5 vol% SrTiO3@SiO2 platelets, Figure S2: Cross-section SEM images of composites filled with 4 vol% SrTiO3@SiO2 platelets, Figure S3: (a) Dielectric constant, (b) dielectric loss as a function of frequency of PVDF and composites filled with SrTiO3 platelets, Figure S4: Unipolar electric displacement–electric fields (D–E) loops at varied electric fields of (a) PVDF and (b–d) composites filled with SrTiO3@SiO2 platelets, Figure S5: Charged energy density at varied electric fields of PVDF and composites filled with SrTiO3 platelets, Figure S6: Efficiency at varied electric fields of PVDF and composites filled with SrTiO3 platelets, Figure S7: Leakage current density at varied electric fields of PVDF and composites filled with SrTiO3 platelets.
