# Supplementary material for: High Breakdown Strength and Energy Storage Density in Aligned SrTiO3@SiO2 Core–Shell Platelets Incorporated Polymer Composites
Source: Membranes (Basel). 2021 Sep 30;11(10):756. doi: 10.3390/membranes11100756 (PMC8541088; doi:10.3390/membranes11100756)
Supplement: Supplementary file 1 [file membranes-11-00756-s001.zip › membranes-1389285-supplementary.pdf]

High breakdown strength and energy storage density in aligned $SrTiO_3@SiO_2$ core-shell platelets incorporated polymer composites

Jie Chen[1], Xiaoyong Zhang [1], Xiao Yang[2], Chuanyang Li[3], Yifei Wang[3,*],Weixing Chen[1]

1 Shaanxi Key Laboratory of Optoelectronic Functional Materials and Devices, School of Materials Science and Chemical Engineering, Xi'an Technological University, Xi'an 710032, China; chenjie@xatu.edu.cn
2 School of Electrical and Electronic Engineering, North China Electric Power University, Beijing, 102206, China; yangxiao@ncepu.edu.cn
3 Electrical Insulation Research Center, Institute of Materials Science, University of Connecticut, Storrs, Connecticut 06269, USA; chuanyang.li@uconn.edu
* Correspondence: y.wang@uconn.edu

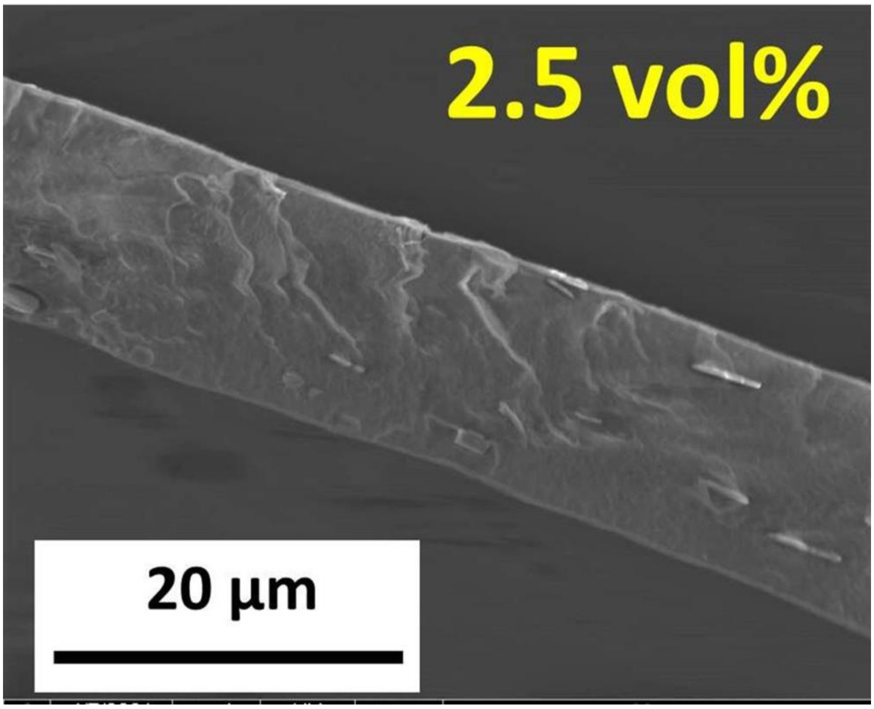

Figure S1. Cross-section SEM images of composites filled with 2.5 vol% $SrTiO_3@SiO_2$ platelets.

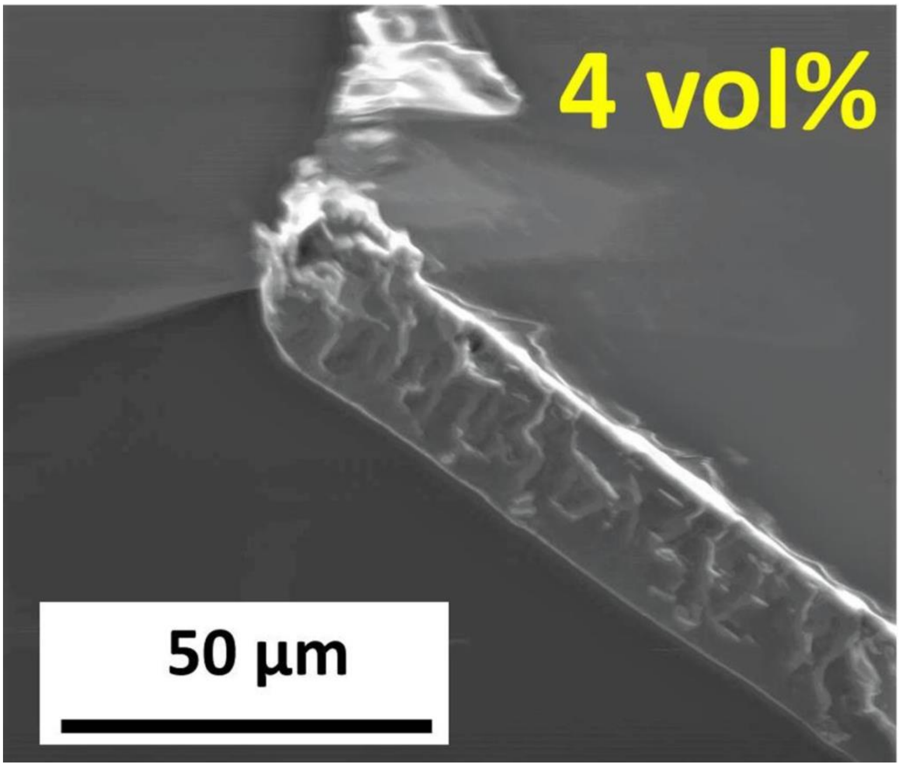

Figure S2. Cross-section SEM images of composites filled with 4 vol% $SrTiO_3$@SiO_2 platelets.

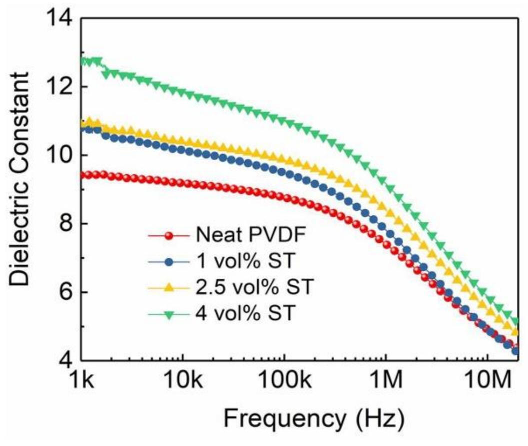

(**a**)

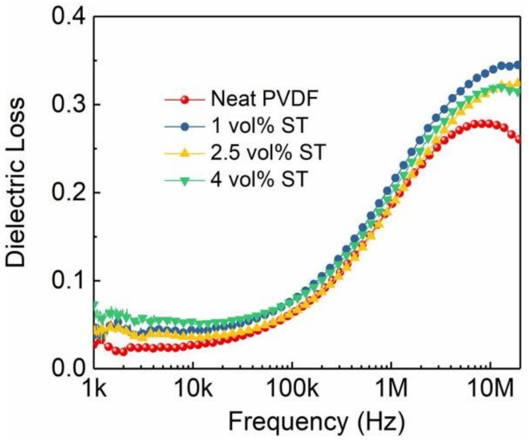

(**b**)

Figure S3. (**a**) Dielectric constant, (**b**) dielectric loss as a function of frequency of PVDF and composites filled with $SrTiO_3$ platelets.

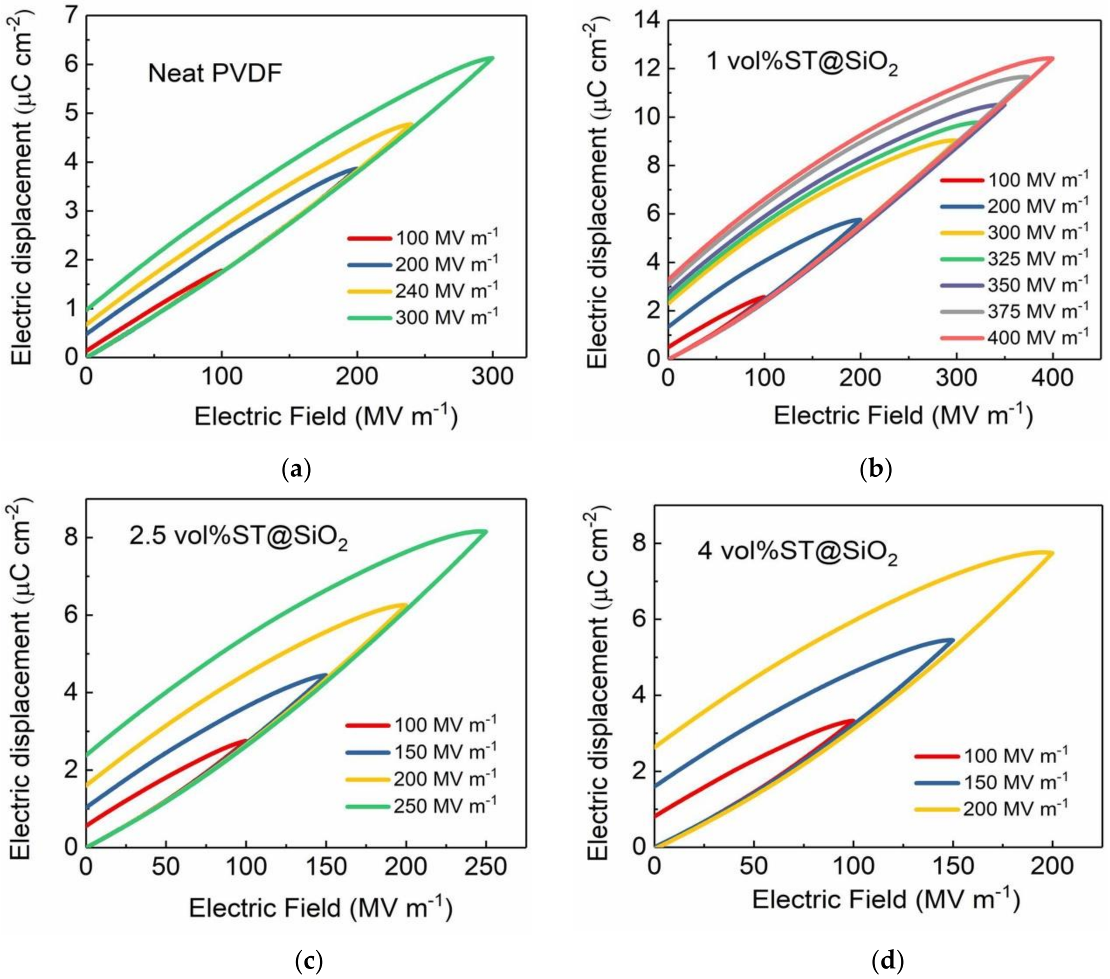

Figure S4. Unipolar electric displacement–electric fields (*D*–*E*) loops at varied electric fields of (**a**) PVDF and (**b-d**) composites filled with $SrTiO_3@SiO_2$ platelets.

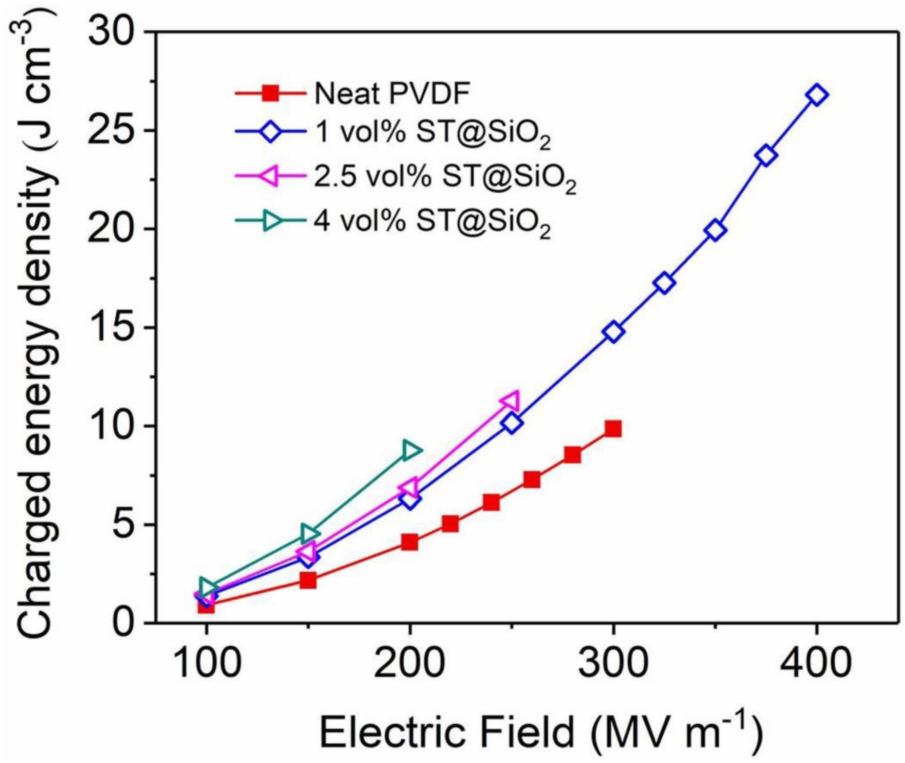

Figure S5. Charged energy density at varied electric fields of PVDF and composites filled with $SrTiO_3$ platelets.

.

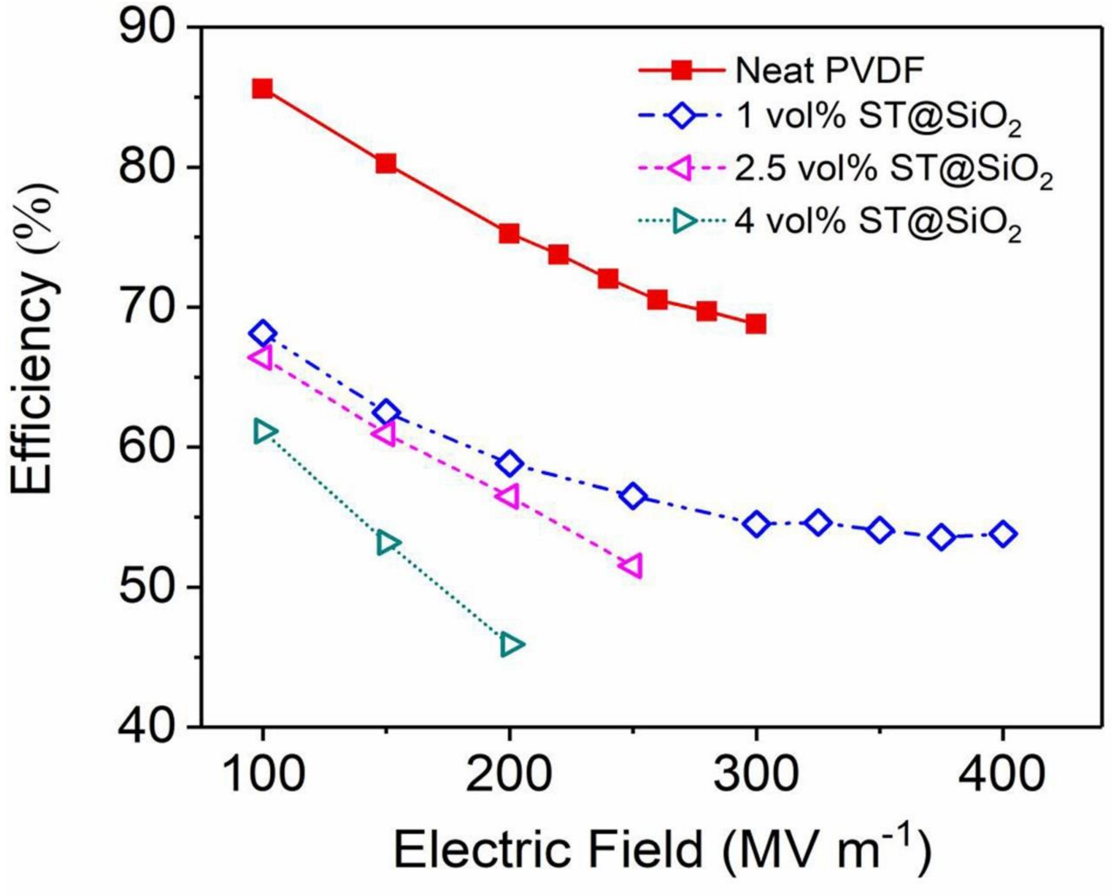

Figure S6. Efficiency at varied electric fields of PVDF and composites filled with $SrTiO_3$ platelets.

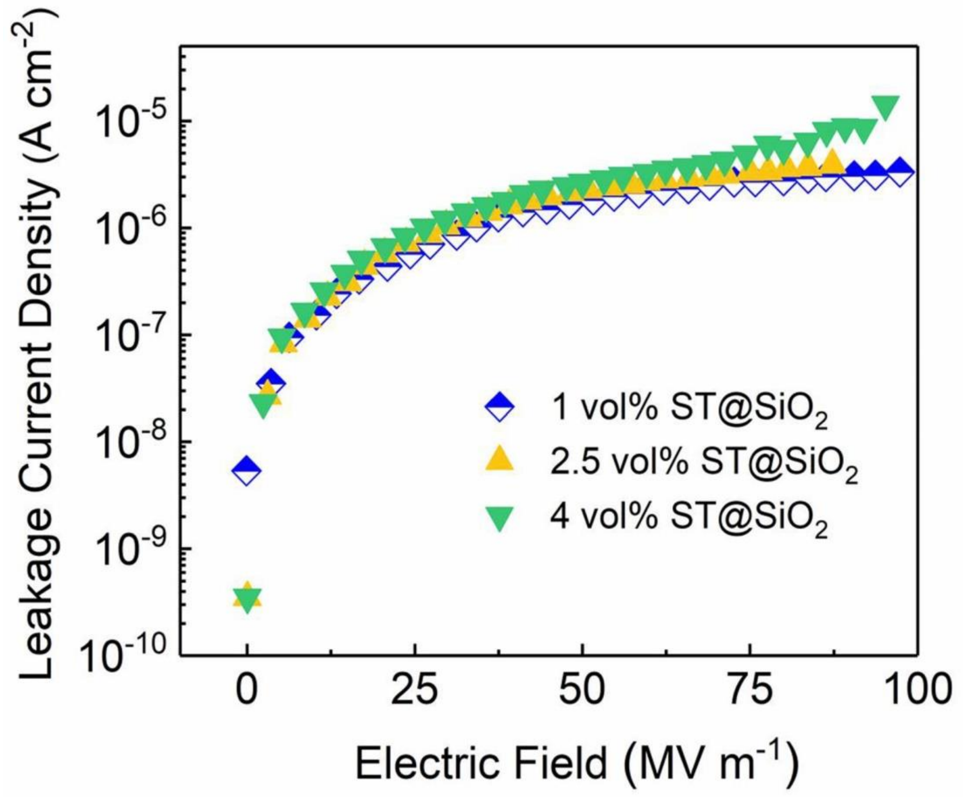

Figure S7. Leakage current density at varied electric fields of PVDF and composites filled with $SrTiO_3$ platelets.
